# LncRNA-miRNA-mRNA regulatory networks in skin aging and therapeutic potentials

**DOI:** 10.3389/fphys.2023.1303151

**Published:** 2023-10-10

**Authors:** Sungchul Kim

**Affiliations:** Center for RNA Research, Institute for Basic Science, Seoul, Republic of Korea

**Keywords:** skin aging, CeRNA (lncRNA-miRNA-mRNA), lncRNA, miRNA, skin wound healing

## Abstract

Skin aging is a complex process influenced by intrinsic and extrinsic factors. Although dermatology offers advanced interventions, molecular mechanisms in skin aging remain limited. Competing endogenous RNAs (ceRNAs), a subset of coding or non-coding RNAs, regulate gene expression through miRNA competition. Several ceRNA networks investigated up to now offer insights into skin aging and wound healing. In skin aging, RP11-670E13.6-miR-663a-CDK4/CD6 delays senescence induced by UVB radiation. Meg3-miR-93-5p-epiregulin contributes to UVB-induced inflammatory skin damage. Predicted ceRNA networks reveal UVA-induced photoaging mechanisms. SPRR2C sequesters miRNAs in epidermal aging-associated alteration of calcium gradient. H19-miR-296-5p-IGF2 regulates dermal fibroblast senescence. PVT1-miR-551b-3p-AQP3 influences skin photoaging. And bioinformatics analyses identify critical genes and compounds for skin aging interventions. In skin wound healing, MALAT1-miR-124 aids wound healing by activating the Wnt/β-catenin pathway. Hair follicle MSC-derived H19 promotes wound healing by inhibiting pyroptosis. And the SAN-miR-143-3p-ADD3 network rejuvenates adipose-derived mesenchymal stem cells in wound healing. Thus, ceRNA networks provide valuable insights into the molecular underpinnings of skin aging and wound healing, offering potential therapeutic strategies for further investigation. This comprehensive review serves as a foundational platform for future research endeavors in these crucial areas of dermatology.

## 1 Introduction

Skin aging, scientifically referred to as cutaneous aging, is a process characterized by the intricate interplay of intrinsic (internal) and extrinsic (external) factors (Gilchrest, 1989; Fisher et al., 2002; Helfrich et al., 2008). It results in a spectrum of discernible signs, including wrinkles, diminished elasticity, age spots, hyperpigmentation, dryness, and more. Importantly, skin aging’s progression varies based on genetics, lifestyle, and environmental influences, making it a highly individualized journey (McCullough and Kelly, 2006; Baumann, 2007). Intrinsic factors encompass genetic predispositions and the natural aging process, while extrinsic factors include cumulative sun exposure, lifestyle choices, and environmental stressors. While skin aging is an inevitable part of life, a proactive approach can significantly mitigate its effects and promote healthier, more youthful-looking skin (Farage et al., 2008). Key preventive measures include rigorous sun protection to shield against harmful ultraviolet radiation (Green et al., 2011), maintaining a well-balanced diet to provide essential nutrients and hydration (Boelsma et al., 2003), and adhering to tailored skincare routines that cater to individual skin types and concerns. Furthermore, abstaining from harmful habits like smoking and excessive alcohol consumption is pivotal in preserving skin health (Poljsak and Dahmane, 2012; Pierard-Franchimont et al., 2019). Beyond preventive measures, dermatology offers a range of advanced interventions to address specific signs of skin aging. These encompass treatments such as botulinum toxin, dermal fillers, chemical peels, laser therapy and molecular regulation of skin aging-related gene expression networks, each tailored to cater to unique skin aging concerns (Carruthers, 2002; Nikalji et al., 2012; Shahrokh et al., 2019; Huth et al., 2020). Although various dermatological aging prevention strategies have been developed in last years, our understanding of molecular mechanisms in skin cell aging has been still limited.

Competing endogenous RNAs (ceRNAs) are a subset of coding or non-coding RNAs (ncRNAs) that encompass various RNA species, including messenger RNAs (mRNAs), transcribed pseudogenes, expressed 3′-untranslated regions (3′-UTRs), long non-coding RNAs (lncRNAs), viral noncoding RNAs, genomic viral RNAs and circular RNAs (circRNAs) (Salmena et al., 2011; Tay et al., 2014; Deniz and Erman, 2017). These RNA molecules compete for a common pool of microRNAs (miRNAs) within the cell. MiRNAs are short RNA molecules that play a crucial role in post-transcriptional gene regulation by binding to complementary sequences in target mRNAs, thereby inhibiting their translation or promoting their degradation (Bartel, 2009; Krol et al., 2010; Ha and Kim, 2014). When multiple RNA molecules contain binding sites for the same miRNA, they can compete for the available miRNAs (Ebert et al., 2007). CeRNAs contain miRNA response elements (MREs), which are sequences that can bind to specific miRNAs. When a ceRNA and a target mRNA share MREs for the same miRNA, they can compete for binding to that miRNA (Bosson et al., 2014; Thomson and Dinger, 2016). When a ceRNA sponges or sequesters a miRNA, it prevents the miRNA from binding to and regulating its target mRNA. As a result, the target mRNA’s expression may increase. Such a ceRNA-miRNA-mRNA interaction creates a complex network of post-transcriptional regulation, where changes in the expression levels of one RNA molecule can influence the expression of others in the network.

In this review, we highlight recently characterized ceRNA networks related to skin aging and wound healing (Table 1). These networks shed light on the molecular mechanisms underlying these processes and offer potential therapeutic strategies to regulate skin cell senescence in the future.

**TABLE 1:** LncRNA-miRNA-mRNA networks related to skin aging and wound healing.

lncRNA	miRNA	mRNA	Context	References
1. Studies in skin aging				
RP11-670E13.6	miR-663a	CDK4/CD6	UVB-induced skin photoaging *in vitro*	Li et al. (2019)
Meg3	miR-93-5p	Epiregulin	UVB-induced inflammatory skin damage *in vitro* and *in vivo*	Zhang et al. (2019)
BICD1-1:1	miR-146a-5p	Not specified	UVA-induced skin photoaging, bioinformatics prediction	Lin et al. (2021)
LPHN3-8:1				
SLC9A11-6:1				
CENPK-2:1				
PRKAR1A-5:2				
SPRR2C	miR-542-5p	Not specified	Epidermal aging-associated alteration of calcium gradient *in vitro* and *in vivo*	Breunig et al. (2021)
	miR-125a			
	miR-135a-5p			
	miR-196a-5p			
	miR-491-5p			
	miR-552-5p			
H19	miR-296-5p	IGF2	Dermal fibroblast senescence *in vitro*	Tang et al. (2022)
PVT1	miR-551b-3p	AQP3	Skin photoaging *in vitro*	Tang et al. (2023)
Not specified	Not specified	Not specified	Skin aging, bioinformatics screening analysis	Xiao et al. (2023)
Comprehensive study for potential ceRNA-PPI networks				
2. Studies in skin wound healing				
MALAT1	miR-124	Not specified	H_2_O_2_-induced wound healing *in vitro*	He et al. (2020)
H19	Not specified	Not specified	Diabetic skin wound healing *in vitro* and *in vivo*	Yang et al. (2023)
	Related to NLRP3 inflammasome pathway			
SAN	miR-143-3p	ADD3	Aged adipose tissue stem cells and wound healing *in vitro* and *in vivo*	Xiong et al. (2023)

Abbreviations: CDK4, cyclin-dependent kinase 4; CD6, cluster of differentiation 6; BICD1, BICD, cargo adaptor 1; LPHN3, Latrophilin 3; SLC9A11, sodium/hydrogen exchanger 11; CENPK, centromere protein K; PRKAR1A protein kinase cAMP-dependent type I regulatory subunit alpha; UV, ultraviolet; SPRR2C, small proline rich protein 2C; IGF2, insulin like growth factor 2; PVT1, plasmacytoma variant translocation 1; AQP3, aquaporin-3; PPI., protein-protein interaction; NLRP3, NLR, family pyrin domain containing 3; SAN, senescence-associated noncoding RNA; ADD3, adducin 3.

### 1.1 CeRNA networks in skin aging

#### 1.1.1 LncRNA RP11-670E13.6-miR-663a-CDK4/CD6 network in UVB-induced skin photoaging

In 2019, Li et al. (2019) conducted an inaugural investigation into ceRNA networks in the context of skin aging. This pioneering study aimed to elucidate the intricate interactions involving lncRNA RP11-670E13.6 within cellular frameworks, particularly its engagement with miRNAs and other cellular constituents. The primary objective was to discern how RP11-670E13.6 functions to safeguard dermal fibroblasts from entering a state of cellular senescence, a process expedited by exposure to ultraviolet B (UVB) radiation, a known accelerator of skin aging (Cav et al., 2017; Lee et al., 2021).

Notably, the study’s findings highlighted a critical interaction between RP11-670E13.6 and a protein known as heterogeneous nuclear ribonucleoprotein (hnRNP) H. This interaction emerged as a pivotal component of the regulatory mechanism responsible for delaying cellular senescence. Specifically, RP11-670E13.6 was observed to act as a sponge for miRNA-663a, thereby modulating the derepression of key factors such as Cdk4 and Cdk6. This modulation, in turn, contributed to the postponement of cellular senescence in dermal fibroblasts subjected to UV irradiation-induced skin photoaging. Furthermore, RP11-670E13.6 exhibited an additional role in facilitating the repair of DNA damage, a crucial process for maintaining cellular integrity. This function was achieved through the upregulation of ATM and γH2A.X levels. Additionally, the study provided evidence that hnRNP H physically interacted with RP11-670E13.6 and exerted a regulatory influence by blocking its expression. This observation suggests that hnRNP H holds promise as a potential therapeutic target in the context of interventions aimed at mitigating skin photoaging.

#### 1.1.2 LncRNA Meg3-miR-93-5p-epiregulin network in UVB-induced inflammatory skin damage

Zhang et al. (2019) delved into the intricate mechanisms underpinning inflammatory skin damage resulting from exposure to UVB radiation, since UVB-irradiation on murine dorsal skin tissues and fibroblasts actually induce inflammation and tissue damage (Afaq et al., 2003; Cav et al., 2017; Her et al., 2019; Lee et al., 2021). Employing a comprehensive ceRNA network analysis, the research team unveiled a pivotal interaction involving lncRNA Meg3, a specific miRNA termed miR-93-5p, and epiregulin. The miR-93-5p was identified as a target susceptible to sequestration by Meg3. Epiregulin, a 46-amino acid protein belonging to the epidermal growth factor (EGF) family of peptide hormones (Riese and Cullum, 2014), has previously been implicated in orchestrating the inflammatory response triggered by UVB radiation exposure (Zha et al., 2019). Through its ceRNA regulatory function, Meg3 played a significant role in upregulating the expression of epiregulin. This upregulation was closely associated with the activation of a pronounced inflammatory response within the skin, culminating in the development of skin lesions and damage. The study’s findings shed light on the Meg3-miR-93-5p-epiregulin axis as a pivotal contributor to the pathogenesis of skin lesions induced by UVB radiation exposure, providing valuable insights into the molecular mechanisms underlying inflammatory skin damage in this context.

#### 1.1.3 Prediction of novel lncRNA-miRNA-mRNA networks in UVA-induced skin photoaging

Lin et al. (2021) conducted an intriguing study in 2021 aimed at predicting lncRNA-miRNA-mRNA networks in human skin photoaging, particularly focusing on the effects of ultraviolet A (UVA) radiation exposure. In this comprehensive investigation, human skin samples subjected to UVA radiation were analyzed using high-throughput sequencing and advanced bioinformatics tools for a thorough examination of miRNA, lncRNA, and mRNA expression profiles. The study revealed the differential expression of 34 miRNAs and their potential interactions with specific lncRNAs.

Notably, an exploration of regulatory networks highlighted the potential impact of signal transduction pathways, including the TNF signaling pathway, thyroid hormone signaling pathway, and lysosome-related processes, following UVA irradiation. Furthermore, miR-146a-5p emerged as a key player, with experimental validation confirming its downregulation post-UVA irradiation. Of particular interest, the study proposed potential interactions between miR-146a-5p and several lncRNAs, namely, BICD1-1:1, LPHN3-8:1, SLC9A11-6:1, CENPK-2:1, and PRKAR1A-5:2. While further validation of this network is warranted, it suggests a potentially crucial upstream regulatory mechanism in the context of dermal UVA-induced photoaging.

LncRNA SPRR2C-mediated miRNA sequestration in epidermal aging-associated alteration of calcium gradient.

In a recent study by Breunig et al. (2021) conducted in 2021, the researchers investigated the adaptive mechanisms of the epidermis in response to altered calcium levels within various skin layers, including the stratum granulosum, the outermost stratum spinosum, and the stratum basale. This investigation shed light on how epidermal cells, particularly keratinocytes, regulate their response to calcium-induced inhibition of cell division by modulating the expression of specific miRNAs. They revealed that several miRNAs, including miR-542-5p, miR-125a, miR-135a-5p, miR-196a-5p, miR-491-5p, and miR-552-5p, exhibited altered expression levels in response to calcium-induced signals. Importantly, these miRNAs were identified as potential sequestration targets of the lncRNA SPRR2C. Through its sponge mechanism, SPRR2C was shown to modulate the levels of these miRNAs, thereby influencing the calcium-induced processes associated with epidermal aging.

#### 1.1.4 LncRNA H19-miR-296-5p-IGF2 axis in senescence of human dermal fibroblasts

Tang et al. (2022) investigated into the role of lncRNA H19 in human dermal fibroblasts (HDFs) concerning cellular viability and senescence. Their research led to the identification of miR-296-5p as a significant player in the regulation of these processes through a comparative analysis of young and aging skin samples. Further exploration revealed that miR-296-5p critical in the context of skin aging exerts its influence by targeting IGF2 mRNA among a pool of three mRNA candidates, IGF2, ACTN1, and ARID3B.

Notably, IGF2 was shown to activate the PI3K/mTOR/AWP3 signaling pathway, leading to the upregulation of AQP3 that plays a critical role in skin aging (Li et al., 2010; Qin et al., 2011; Bollag et al., 2020), which in turn suppressed cell viability and was associated with the senescence of HDFs. Moreover, this study proposed lncRNA H19 as a ceRNA for miR-296-5p, implying its role in sequestering and modulating the activity of miR-296-5p. This finding collectively positions lncRNA H19 as a novel molecular target for potential therapeutic interventions aimed at delaying the skin aging process.

#### 1.1.5 LncRNA PVT1-miR −551b-3p-AQP3 network in skin photoaging

In a recent study by Tang et al. (2023), the focus was directed towards elucidating the regulatory mechanisms underlying skin photoaging in HDFs mediated by lncRNAs. They employed *in silico* analysis to identify photoaging-related genes, followed by the screening of differentially expressed lncRNAs and miRNAs to establish ceRNA interaction networks. Among the genes examined, AQP3 emerged as a noteworthy candidate (Jing et al., 2016), exhibiting a negative correlation with aging in HDFs within one of the datasets. Subsequent experiments validated the role of AQP3 in enhancing HDF viability and mitigating senescence, primarily by impeding the ERK/p38 MAPK signaling pathway.

Intriguingly, miR-551b-3p, identified as one of the upstream miRNAs of AQP3 through the ENCORI database, was found to be significantly upregulated in senescent HDFs, suggesting its involvement in the aging process. Furthermore, the study predicted potential upstream lncRNA regulators of miR-551b-3p and identified PVT1 as a downregulated candidate in senescent HDFs. Mechanistically, PVT1 was demonstrated to function as a sponge for miR-551b-3p in senescent HDFs, effectively suppressing its expression through seed-mediated base-pairing. These findings collectively shed light on the intricate regulatory networks governing skin photoaging in HDFs.

#### 1.1.6 Bioinformatics analysis of lncRNA-miRNA-mRNA networks in skin aging

Xiao et al. (2023) advanced computational and bioinformatics methodologies, which were harnessed to comprehensively analyze extensive datasets containing genetic and molecular information pertinent to the intricate phenomenon of skin aging. They meticulously examined two distinct gene expression datasets, namely, GSE55118 and GSE72264, which are particularly pertinent to skin aging processes. This analysis identified a curated selection of 169 mRNAs, 27 miRNAs, and 50 lncRNAs that exhibited close associations with skin aging within co-expression networks. As a consequential outcome of this analysis, the study spotlighted ten hub genes, which include AQP4, TRPM8, TBR1, NTSR2, MPPED1, BARHL2, PAX9, CPN1, CES3, and CHGB. These hub genes were determined to play pivotal roles in orchestrating protein-protein interactions (PPIs) relevant to the progression or potential reversal of skin aging (Jing et al., 2016; Bicakci et al., 2017; Ikarashi et al., 2017; Owasil et al., 2020; Thapa et al., 2021; Chen et al., 2022a; Chen et al., 2022b), underscoring their significance in the intricate biological processes involved.

Furthermore, this study extended its inquiry to identify ten potential compounds with the capacity to alleviate skin aging. These compounds, including tretinoin (Bergstrom, 2009), pifithrin (Marsolais et al., 2007), selamectin (Bozzatto et al., 2014), entinostat (Jiang et al., 2023), bretazenil (Guldner et al., 1995), syringic-acid (Ha et al., 2018; Ren et al., 2019; Abd-Allah et al., 2023), BRD-K96475865, emedastine (Murota et al., 2008), abacavir (Chuang and Chen, 2018), and rotenone (da Cruz et al., 2023), were rigorously validated through molecular docking analysis with AQP4, which was ranked the core in the PPI analysis. Such computational insights into promising compounds hold substantial promise for the development of interventions aimed at mitigating the effects of skin aging. In summation, the comprehensive dataset and findings generated by this study constitute a valuable resource that not only deepens our understanding of the molecular underpinnings of skin aging but also serves as a foundational platform for future investigations in this crucial area of research.

### 1.2 CeRNA networks in skin wound healing

#### 1.2.1 LncRNA MALAT1-miR-124 network in H_2_O_2_-induced wound healing

He et al. (2020) investigated the intricate mechanisms underlying wound healing, with a particular focus on extracellular vesicles (exosomes) derived from adipose-derived stem cells (ADSCs) harboring the lncRNA MALAT1. This research unveiled a specific miRNA, miR-124, as a key target of MALAT1 in HaCaT and HDF cells. Importantly, MALAT1-containing ADSC-Exos were found to play a crucial role in the activation of the Wnt/β-catenin signaling pathway. Through this activation and the concurrent targeting of miR-124, MALAT1-containing ADSC-Exos were demonstrated to facilitate and expedite the wound healing process induced by hydrogen peroxide (H_2_O_2_). These findings offer novel insights into potential therapeutic approaches for enhancing wound healing in human normal subcutaneous adipose tissues.

#### 1.2.2 MSC-comprised exosomal lncRNA H19 for NLRP3 regulation in diabetic skin wound healing

Yang et al. (2023) has further explored the potential role of mesenchymal stem cell (MSC)-derived exosomes containing lncRNA H19 in the context of skin wound healing in individuals with diabetes. This study encompassed a series of experiments involving the use of human immortalized keratinocyte cell line HaCaT cells and murine models. Their findings revealed that exosomes derived from hair follicle MSCs, which encapsulated lncRNA H19, exhibited the capacity to augment cell proliferation and migration. This effect was attributed to the inhibition of pyroptosis, a form of programmed cell death, achieved by suppressing the activation of the NLRP3 inflammasome. These observations were consistent both *in vitro* experiments conducted with HaCaT cells and *in vivo* studies using murine models. Consequently, these findings suggest that lncRNA H19 holds promise as a potential therapeutic candidate for the repair of skin wounds in individuals afflicted with diabetes.

#### 1.2.3 LncRNA SAN-miR-143-3p-ADD3 network in aged adipose tissue stem cells and wound healing

Clinical applications involving cell-based wound healing hold immense therapeutic promise across a spectrum of medical contexts. Adipose-derived mesenchymal stem cells (ASCs) have garnered considerable attention for their utility in promoting wound healing (Jo et al., 2021). However, the therapeutic potential of ASCs appears to be compromised with aging, necessitating a concerted effort to mitigate the senescence-associated decline in their efficacy. Addressing this critical concern, a recent study by Xiong et al. (2023) introduced an innovative approach for rejuvenating ASCs by leveraging the regulatory properties of a lncRNA known as senescence-associated noncoding RNA (SAN). In this study, they delved into the pivotal role played by SAN as a ceRNA against miR-143-3p, a known regulator of ASC senescence through its targeting of ADD3 mRNA, as elucidated in prior research (Deacon et al., 2010). These findings suggest that SAN, by acting as a molecular sponge for miR-143-3p, exerts control over the senescence-related processes in ASCs. While acknowledging the need for further *in vitro* and *in vivo* validations and in-depth mechanistic investigations, the study underscores the significance of the lncRNA SAN-miR-143-3p-ADD3 network in governing ASC senescence. Importantly, this research sheds light on the potential of lncRNAs as invaluable therapeutic tools for effectively managing the aging-related challenges encountered in cell-based wound healing strategies.

## 2 Concluding remark

In the relentless pursuit of elucidating the intricate molecular mechanisms underpinning the processes of skin aging and wound healing, the burgeoning domain of ceRNA networks has emerged as a fertile ground yielding promising insights and potential therapeutic avenues. CeRNAs, constituting a subset of non-coding RNAs, have conspicuously ascended as pivotal orchestrators of gene expression regulation, exerting their influence through intricate miRNA-mediated competition. This review showcases a compendium of ceRNA networks, the elucidation of which collectively contributes to the enhancement of our comprehension regarding the molecular underpinnings of skin aging and wound healing. These revelations offer a solid foundation upon which future research and therapeutic strategies can be erected.

It's important to note that the development of ceRNA-based therapeutics is still in its early stages, and many challenges need to be addressed, including delivery methods, specificity, and potential off-target effects. Additionally, the success of ceRNA-based interventions by targeting central nodes in context would depend on a thorough understanding of the ceRNA networks involved in the particular skin aging process or condition of interest. Further molecular validations and clinical studies are needed to determine the feasibility and effectiveness of these approaches compared to miRNA or mRNA-based regulation.

In the last decade, there have been lots of experimentally validated supports for the ceRNA-miRNA-mRNA networks that affect complexed cellular processes, such as cancer biology, cellular development, and host cell regulation by viruses (Tay et al., 2014; Thomson and Dinger, 2016; Xu et al., 2022). Likewise, the multifaceted potential inherent in ceRNA networks augurs well for their role as a versatile and dynamic toolset in the relentless quest to promote the attainment of healthier, more youthful skin and to bolster the efficacy of wound healing processes. It is imperative to note, however, that substantial terrain remains uncharted in the realm of ceRNA networks, promising a fertile landscape for further scientific exploration. In the foreseeable future, fortified by the knowledge gleaned from these endeavors, individuals may find themselves endowed with a diverse molecular arsenal, empowering them to adopt proactive approaches in the stewardship and rejuvenation of their skin, thereby enabling the realization of the full spectrum of their skin’s innate health and aesthetic potential.
